# Mutational profile of primary clear cell renal cell carcinoma predicts recurrence and potential candidacy for adjuvant immune checkpoint inhibition

**DOI:** 10.12688/f1000research.136087.2

**Published:** 2024-06-14

**Authors:** Panagiotis J. Vlachostergios, Maria Papathanassiou, Maria Anagnostou, Eleni Thodou, Ioannis Tamposis, Lampros Mitrakas, Ioannis Zachos, George K. Koukoulis, Maria Samara, Vassilios Tzortzis

**Affiliations:** 1 Urology, University of Thessaly, Faculty of Medicine, School of Health Sciences, University Hospital of Larissa, Larissa, Thessalia, Greece; 2 Medical Oncology, IASO Thessalias Hospital, Larissa, Thessalia, Greece; 3 Hematology & Medical Oncology, Weill Cornell Medicine, New York, New York, USA; 4 Pathology, University of Thessaly, Faculty of Medicine, School of Health Sciences, Larissa, Greece; 5 Computer Science and Biomedical Informatics, University of Thessaly, Lamia, Greece

**Keywords:** clear cell, kidney cancer, renal cell carcinoma, recurrence, immune checkpoint inhibition, genomics, mutational profile, next generation sequencing

## Abstract

**Background:**

The risk of recurrence after nephrectomy for primary clear cell renal cell carcinoma (ccRCC) is estimated in daily practice solely based on clinical criteria. The aim of this study was to assess the prognostic relevance of common somatic mutations with respect to tumor aggressiveness and outcomes of ccRCC patients after definitive treatment.

**Methods:**

Primary tumors from 37 patients with ccRCC who underwent radical nephrectomy were analyzed for presence of somatic mutations using a 15-gene targeted next-generation sequencing (NGS) panel. Associations to histopathologic characteristics and outcomes were investigated in the study cohort (n=37) and validated in The Cancer Genome Atlas (TCGA) ccRCC cohort (n=451).

**Results:**

*VHL* was the most frequently mutated gene (51%), followed by *PBRM1* (27%), *BAP1* (13%), *SETD2* (13%), *KDM5C* (5%), *ATM* (5%), *MTOR* (5%), and *PTEN* (3%). One-third of patients did not have any somatic mutations within the 15-gene panel. The vast majority of tumors harboring no mutations at all or VHL-only mutations (51%) were more frequently of smaller size (pT1-2) and earlier stage (I/II), whereas presence of any other gene mutations in various combinations with or without *VHL* was enriched in larger (pT3) and higher stage tumors (III) (p=0.02). No recurrences were noted in patients with unmutated tumors or *VHL*-only mutations as opposed to three relapses in patients with non- *VHL* somatic mutations (p=0.06). Presence of somatic mutations in *PBRM1, BAP1, SETD2, KDM5C, ATM, MTOR*, or *PTEN* genes in 451 TCGA ccRCC patients was associated with a significantly shorter disease-free survival (DFS) compared to those with unaltered tumors (q=0.01).

**Conclusions:**

Preliminary findings from this ongoing study support the prognostic value of non- *VHL* mutations including *PBRM1, BAP1, SETD2, KDM5C, ATM, MTOR*, and *PTEN* in primary ccRCC tumors as surrogates of earlier recurrence and potential selection for adjuvant immune checkpoint inhibition.

## Introduction

Renal cell carcinoma (RCC) is a heterogenous group of kidney cancers originating from the nephron. ^1^ Renal cell carcinoma ranks among the ten most frequently diagnosed malignancies worldwide, with an estimated 400.000 new diagnoses and more than 170.000 deaths annually. ^2^^,^^3^ Clear cell RCC (ccRCC) is the most common RCC subtype, accounting for about 70-75% of cases and has a distinct molecular profile. ^1^^,^^4^

Localized ccRCC is treated with surgical resection, either partial or radical nephrectomy. Nevertheless, one-third of these patients experience recurrence. ^5^ So far, ccRCC recurrence, disease progression and mortality are being predicted using clinicopathological criteria. ^6^ While various recurrence models have been proposed, they only marginally outperformed standard staging. ^7^ Further, they demonstrate statistically significant variability in their predictive ability over time, rendering implementation into clinical practice and clinical trial design challenging. ^7^

Immune checkpoint inhibition with the use of pembrolizumab, a monoclonal antibody against programmed death-1 (PD-1), is approved as adjuvant therapy for patients with resected ccRCC who have a high risk of recurrence. ^8^ This was based on results of a phase III randomized double-blind study comparing pembrolizumab with placebo, which demonstrated a significant improvement in disease-free survival (DFS). ^9^ Patient selection criteria for a high risk of recurrence included tumor stage II with nuclear grade 4 or sarcomatoid differentiation, tumor stage III or higher, regional lymph-node metastasis, or stage M1 without evidence of disease after combined nephrectomy and metastasectomy either concurrently or within a year from primary tumor resection. ^9^

What currently remains an unmet need is the ability to predict which patients with ccRCC will relapse using a single or composite molecular biomarker that would be more directly related with tumor biology.

In this ongoing prospective study, we examined the mutational profile of patients with non-metastatic ccRCC who underwent nephrectomy, followed by observation or adjuvant immunotherapy with pembrolizumab depending on established clinical and histopathological criteria. We studied associations of mutated genes with high-risk features and assessed the prognostic relevance of somatic mutations with regard to DFS after nephrectomy with or without adjuvant immunotherapy.

## Methods

### Ethical considerations

The study was conducted in accordance with the Declaration of Helsinki and approved by the Institutional Review Board and Ethics Committee of Faculty of Medicine, University of Thessaly (3214/29.07.2016) on 29 July 2016.

### Study design

This was a prospective single-center cohort study of patients with a diagnosis of ccRCC who underwent radical nephrectomy followed by observation or adjuvant immune checkpoint inhibition with pembrolizumab at the University Hospital of Larissa between December 2020 and February 2023. Eligible subjects included patients of ≥18 years of age, with histologically confirmed non-metastatic ccRCC, without prior systemic therapy for RCC. Patients were eligible if they had an intermediate-to-high or high risk of recurrence based on histopathological features including pT2 with grade 4 or sarcomatoid differentiation, pT3 or pT4 with any grade, or any pT and grade with presence of positive lymph nodes (N+). Subjects unable to provide consent, those with low risk of recurrence or subjects receiving steroids at a daily dose above 10mg of prednisone for an active autoimmune or other condition were excluded from the study. The primary endpoint of the study was the percentage of patients without disease recurrence.

### Data collection

Fresh frozen tissue samples were acquired from patients. Tumor tissue was acquired after surgical resection, cut in 5 mg cubes and stored in stabilization solution (RNA later, Thermo Fisher Scientific) at -80 ^o^C freezer, after written informed consent was obtained. DNA extraction and quantification was performed from 5 mg of fresh-frozen tissue. Library preparation was conducted with the use of DNA AmpliSeq for Ion Torrent, with an input of 40 ng DNA per sample. Sample libraries were quantified with the use of Qubit (Thermo-Fisher Scientific) and real-time polymerase chain reaction (PCR), then sequenced on Ion Torrent S5 sequencer, using the Oncomine Kidney panel (Thermo-Fisher Scientific). Library amplification was conducted using the Library PLUS for Ion Torrent kit (Thermo Fisher). Library amplification included enzyme activation at 99 ^o^C for 2 minutes, 16 cycles of denaturation at 99 ^o^C for 15 seconds and annealing and extension steps at 60 ^o^C for 4 minutes and a final hold step at 10 ^o^C. The commercially available Oncomine ^TM^ Kidney Panel (Thermo Fisher Scientific) provided the primer pairs used for library preparation. qPCR was conducted using the Ion Universal Library Quantitation Kit (Thermo Fisher Scientific). qPCR steps include an initial step of incubation at 50 ^o^C for 2 minutes, a polymerase activation step at 95 ^o^C for 2 minutes, 40 cycles of 95 ^o^C for 15 seconds and 60 ^o^C for 1 minute and a final hold step at 10 ^o^C. ^10^

Clinicopathological characteristics recorded for the analysis included patient age, sex, ISUP grade, tumor diameter, T-stage, presence of vascular invasion, presence of sarcomatoid differentiation, presence of necrosis, AJCC stage, and emergence of recurrent disease during follow up. Nephrectomy surgical specimens were reviewed by two independent pathologists from our institution.

A publicly available database, cBioportal for Cancer Genomics (accessed on 24 May 2023), was used to query DNA sequencing data for mutations in a prospective multicenter cohort from The Cancer Genome Atlas (TCGA) including 451 patients with ccRCC (accessed on 24 May 2023).

### Data analysis

The following genes were analyzed for presence of somatic mutations: *ATM, BAP1, KDM5C, MET, MTOR, NF2, PBRM1, PIK3CA, PTEN, SETD2, SMARCB1, TP53, TSC1, TSC2*, and *VHL.* Variant calling was performed using the Ion Reporter Software (Thermo-Fisher Scientific). The Pearson’s Chi squared test was used to determine whether there was a statistically significant difference in clinicopathological characteristics and emergence of recurrence between subgroups of patients with distinct mutational profiles (unmutated or *VHL*-only mutated versus other gene mutations). Time-to-event outcomes (DFS) were estimated using the Kaplan-Meier method. Multiple hypothesis test correction was applied using the Benjamini–Hochberg method. All tests were two-sided, and p and q values ≤0.05 were considered statistically significant. The IBM SPSS v.22 software was used for the analysis.

## Results

### Mutations in non- *VHL* genes are associated with more aggressive disease

We first assessed the frequency of mutations in primary ccRCC tumors. In the discovery cohort (n=37), patients’ clinical and histopathological characteristics are described in Table 1. *VHL* was the most frequently mutated gene (n=19; 51%), followed by *PBRM1* (n=10; 27%), *BAP1* (n=5; 13%), *SETD2* (n=5; 13%), *KDM5C* (n=2, 5%), *ATM* (n=2, 5%), *MTOR* (n=2, 5%), and *PTEN* (n=1, 3%) ( Table 2). Variant types per gene are listed in the data file. ^35^ 11 patients (30%) did not have any somatic mutations within the 15-gene targeted panel.

**Table 1.** Clinical and histopathological characteristics of ccRCC patients (discovery cohort, n=37).

Characteristic	Number (%)
Age	
median (range)	63 (42-87)
Sex	
males	28 (75)
Tumor diameter (cm)	
median (range)	5.6 (1.6 – 15)
Pathological T stage	
T1	21 (57)
T2	6 (16)
T3	10 (27)
ISUP grade	
2	13 (35)
3	15 (41)
4	8 (24)
Vascular invasion	5 (14)
Necrosis	13 (35)
Sarcomatoid differentiation	0
AJCC TNM stage	
I	21 (57)
II	6 (16)
III	10 (27)

**Table 2.** Frequency of mutated genes in primary ccRCC tumors (discovery cohort, n=37).

Gene	Frequency, n (%)
*VHL*	19 (51)
*PBRM1*	10 (27)
*BAP1*	5 (13)
*SETD2*	5 (13)
*KDM5C*	2 (5)
*ATM*	2 (5)
*MTOR*	2 (5)
*PTEN*	1 (3)

Tumors harboring no mutations at all or only *VHL* mutations (n=19, 51%) were associated with smaller size (pT1-2 n=17, 89%) and earlier stage (I/II n=17; 89%), whereas presence of any other gene mutations in various combinations with or without *VHL* was enriched in larger (pT3, n=8; 44%; p=0.02) and more advanced tumors (III, n=8; 44%; p=0.02) ( Table 3). There was also a trend towards higher frequency of ISUP grade, vascular invasion, and necrosis in these tumors ( Table 3).

**Table 3.** Associations between histopathological characteristics and mutational profile (discovery cohort, n=37).

Variable	Mutated genes		P value
	None or *VHL*-only	Non- *VHL*	
Pathological T stage			0.02
T1/T2	17 (89)	8 (44)	
T3	2 (11)	10 (56)	
AJCC TNM stage			0.02
I/II	17 (89)	8 (44)	
III	2 (11)	10 (56)	
ISUP grade			0.93
2/3	15 (79)	14 (78)	
4	4 (21)	4 (22)	
Necrosis			0.64
no	13 (68)	11 (61)	
yes	6 (32)	7 (39)	
Vascular invasion			0.58
no	17 (89)	15 (83)	
yes	2 (11)	3 (17)	

**Figure 1.**
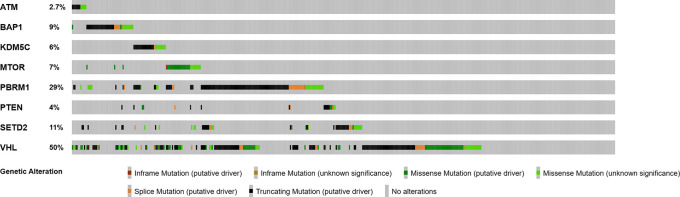
Frequency of mutated genes in primary ccRCC tumors (validation cohort, n=451).

**Figure 2.**
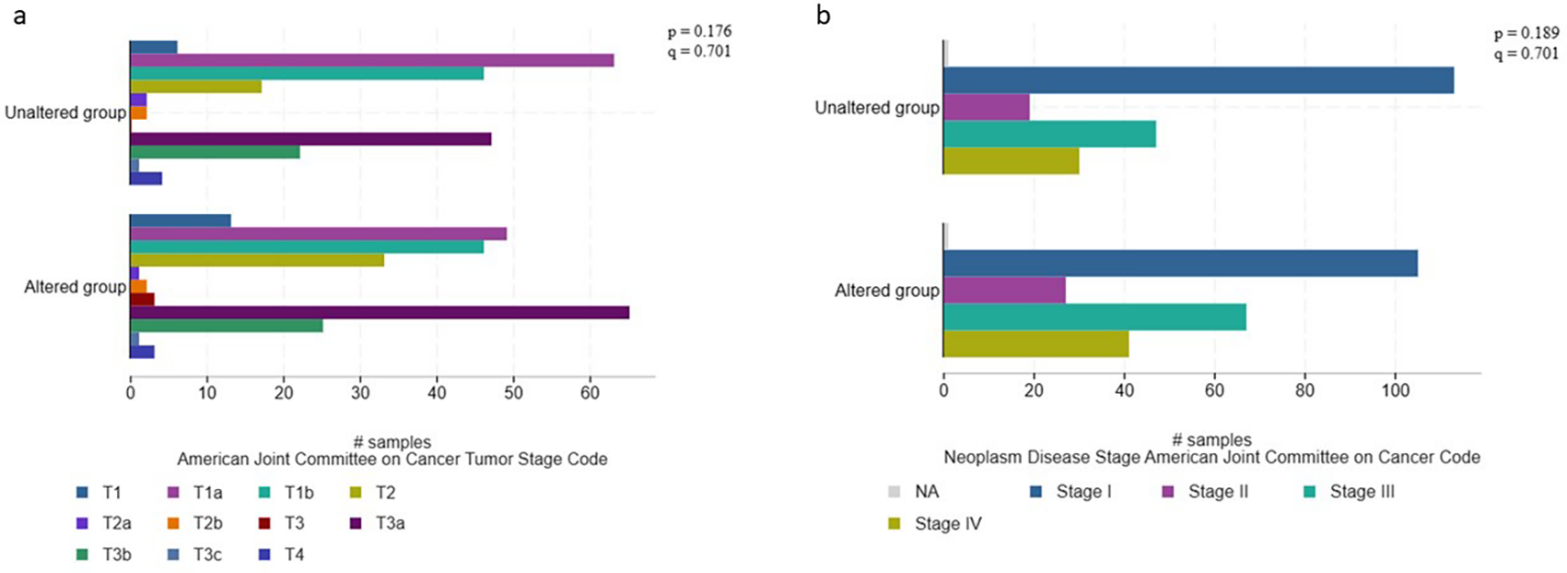
Associations between histopathological characteristics and mutational profile (validation cohort, n=451).

In the validation TCGA cohort (n=451), similar mutations frequencies were noted, including *VHL* in 50% of patients/samples, *PBRRM1* in 29%, *SETD2* in 11%, *BAP1* in 9%, *MTOR* in 7%, *KDM5C* in 6%, *PTEN* in 4%, and *ATM* in 2.7%, respectively ( Figure 1). Larger and higher stage tumors, particularly T3a, T3b, and stage III tended to have a higher frequency of non-VHL mutations (p=0.282; q=0.437) ( Figure 2).

### Mutations in non- *VHL* genes predict recurrence

**Figure 3.**
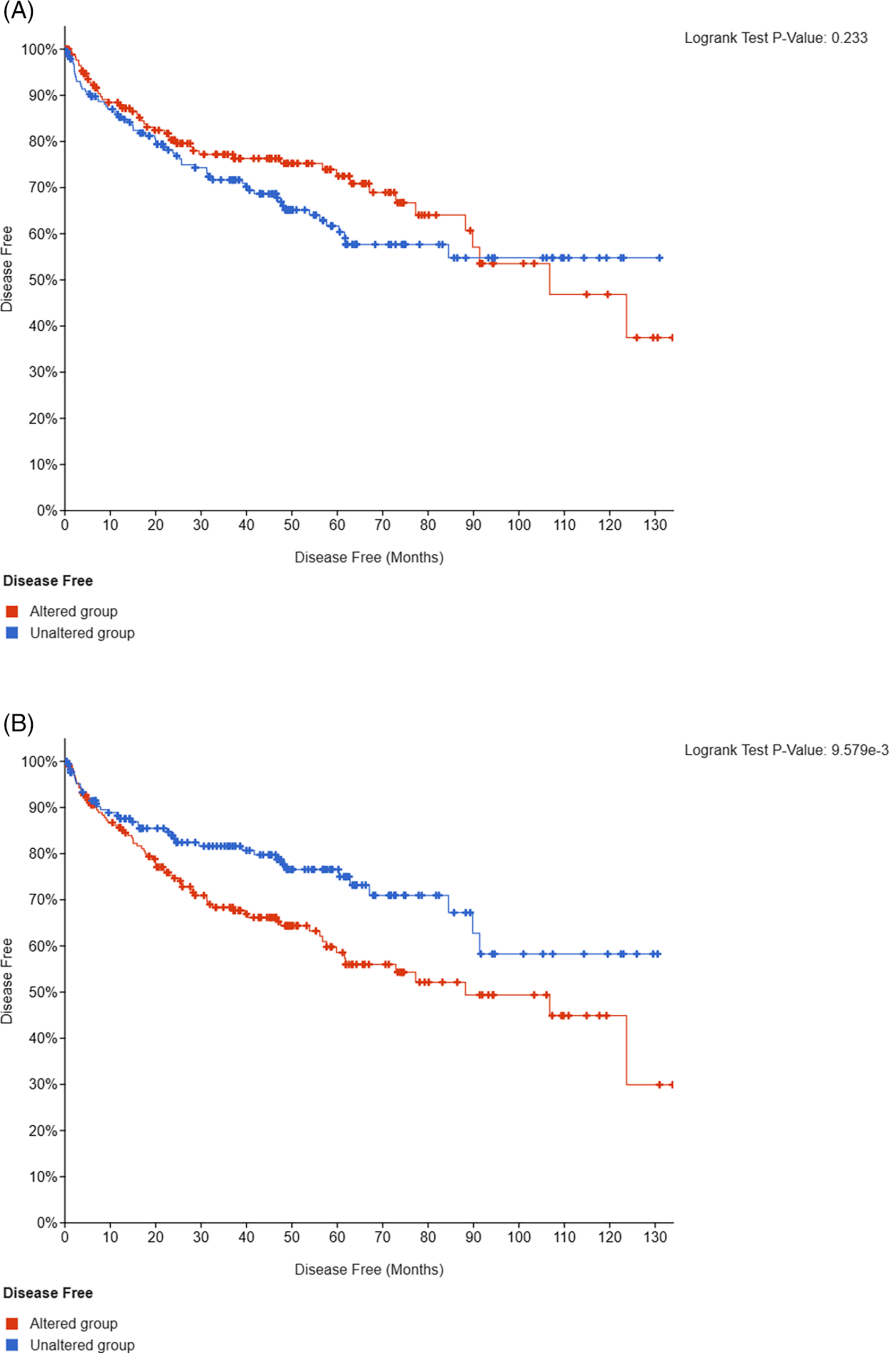
Kaplan-Meier analysis of DFS in patients with unmutated or *VHL*-only versus non- *VHL* mutated tumors (validation cohort, n=451).

No recurrences were noted in patients with unmutated tumors or *VHL*-only mutations whereas three patients (17%) with other somatic mutations relapsed (p=0.06) ( Table 4). Another three patients received and completed adjuvant PD-1 inhibition with pembrolizumab until present, none of whom recurred despite the presence of *PBRM1* and *SETD2* mutations in 2/3 and 1/3, respectively. Presence of somatic mutations in *PBRM1, BAP1, SETD2, KDM5C, ATM, MTOR*, or *PTEN* genes in 451 TCGA ccRCC patients was associated with a significantly shorter DFS compared to those with unaltered tumors (p<0.001; q=0.01) ( Figure 3).

**Table 4.** Associations of mutational profile (none or VHL-only versus non-VHL mutated genes) and disease recurrence.

Outcome	Mutated genes		P value
	None or *VHL*-only	Non- *VHL*	
Recurrence			0.06
no	19 (100)	15 (83)	
yes	0 (0)	3 (17)	

## Discussion

Cancer is a disease caused by the accumulation of genomic alterations. ^11^ This is a multistep process during which certain somatic mutations confer a survival advantage by activating signaling pathways that are associated with several hallmarks of cancer, including proliferative signaling, evasion of growth suppression, resistance to cell death, replicative immortality, angiogenesis, invasion and metastasis, reprogramming of energy metabolism and evasion of immune recognition and destruction. ^12^ Since these biological principles apply to the majority of solid tumors, including RCCs, we hypothesized that the higher the number of pathogenic mutations within a primary renal tumor, the more aggressive clinical behavior could be observed.

The tumor suppressor *VHL* is the most frequently mutated gene in ccRCC and is a major player in renal cell carcinogenesis. However, *VHL* mutations alone are insufficient to drive disease progression, and have no prognostic or predictive value. ^13^^,^^14^ Mounting evidence has revealed an emerging role of other genes, heavily involved in chromatin rearrangement and epigenetic DNA modifications, including *PBRM1, SETD2, BAP1*, and *KDM5C* in ccRCC progression. ^15^^,^^16^ Inactivating mutations or/and low expression of these genes in primary renal tumors have been associated with poor outcomes. ^17^^,^^23^

Interestingly, many of these frequently non- *VHL* genes, including *PBRM1, BAP1, SETD2*, have been investigated in the metastatic setting as potential predictors of response or resistance to immune checkpoint inhibitors (ICIs). For example, mutated *PBRM1* was included in a composite biomarker together with tumor infiltrating lymphocytes (TILs) and absence of necrosis predicting a favorable response to ICIs. ^24^^,^^25^ Likewise, *SETD2* loss results in greater vulnerability to immune checkpoint blockade compared to SETD2-proficient tumors, *in vitro* and *in vivo*. ^26^ *BAP1*-mutated tumors, while they portend a worse prognosis, they are more likely to be PD-L1 positive and demonstrate a more inflamed immune microenvironment suggesting that immune-targeting approaches could benefit these patients. ^27^ Another DNA damage response and repair gene, ATM, was the second most frequently altered gene in ICI-responders with advanced RCC. ^28^ Pancancer analyses of tumors from ICI-treated patients suggested that those with KDM5C alterations have a substantially higher tumor mutational burden (TMB) level and a significantly higher level of CD8+ T cell infiltration and T effector signature which were associated with prolonged OS compared to the wild-type group. ^29^ Expression of PTEN/PI3K/mTOR pathway genes was significantly associated with numerous immune cells and immune-evasive mediators such as *CD274/PD-L1, TGFBR1, CSF1R* and *PDCD1* in patients with ccRCC, ^30^ suggesting that alterations in this pathway could also play a role in shaping these patients’ outcomes after treatment with ICIs.

Our ongoing prospective study examined the mutational profile of patients with ccRCC on primary tumors after nephrectomy followed by observation or adjuvant immunotherapy with pembrolizumab and assessed for associations of mutated genes with high-risk features and DFS. We hypothesized that primary RCC tumors either umutated or harboring VHL mutations might be associated with a more benign clinical course after nephrectomy compared to those with a burden of mutations in other, non-VHL genes.

In this preliminary analysis of the first 37 patients, non- *VHL* mutations, including mutations in *PBRM1, SETD2, BAP1, KDM5C, MTOR, PTEN*, or *ATM* genes in a targeted 15-gene NGS panel were significantly associated with more aggressive histopathological characteristics including larger size, and higher stage. Presence of mutations in any of those genes as opposed to completely unmutated or *VHL*-only mutated tumors was also associated with higher ISUP grade, necrosis, sarcomatoid differentiation, vascular invasion and predicted recurrence. In ccRCC tumors from TCGA, non- *VHL* mutations in the same genes were also associated with high-risk features and predicted a significantly shorter DFS compared to *VHL*-only mutations or complete absence thereof.

Due to the retrospective nature of previous studies and lack of reproducibility, particularly across immunohistochemical assessments ^31^^,^^32^ in an era when observation was the only available modality post-operatively even in high-risk patients, there has been a paucity of data to support testing of these genes as a molecular tool to assist in selection of patients who might benefit from adjuvant therapy.

Our ongoing prospective study addresses this gap by demonstrating that patients who had either no mutation or mutations in the most frequently altered gene, *VHL*, were more likely to have smaller tumors and experienced a more benign course without relapse, compared to those patients with tumors that harbored mutations in other genes, including *PBRM1, BAP1, SETD2, KDM5C, ATM, MTOR*, or *PTEN.* Thus, this study suggests that patients with mutations in these high-risk genes might be more suitable candidates and should be prioritized for post-operative immunotherapy with pembrolizumab, which has demonstrated clinical benefit in PFS and OS and is currently approved as adjuvant treatment. ^33^

Our findings are in line with a previous work with slightly different design, whereby primary RCC tumors were segregated into *VHL*+0, *VHL*+1, *VHL*+2, and *VHL*+≥3 mutations. ^33^ In both the discovery and validation cohorts of the study, those patients with a VHL+0 tumor had longer 5-year DFS and were proposed as candidates for surveillance. Conversely, patients with VHL+2 and VHL+≥3 tumors experienced shorter DFS rates of less than 50% and were deemed candidates for adjuvant therapy. ^34^

Our study was limited by small size and relatively short follow-up of patients. Additionally, the lack of transcriptional or/and epigenetic analyses is another limitation of our study. Nevertheless, presence of an early “signal” of high-risk genes in this preliminary report will be further studied in additional patients being accrued as part of this ongoing prospective study.

Collectively, our study combined with emerging evidence on the genomic landscape of RCC might open new avenues for both prognostication and better selection of a subgroup of patients with RCC that could benefit from adjuvant anti-PD1 immunotherapy.

## Data Availability

figshare: F1000Res_Vlachostergios et al_raw data (subm).xlsx. https://doi.org/10.6084/m9.figshare.23697252.v1. ^23^ Data are available under the terms of the Creative Commons Attribution 4.0 International license (CC-BY 4.0). Data from the validation TCGA cohort analyzed in this study are available from cbioportal.org, http://www.cbioportal.org/study/summary?id=kirc_tcga.
