# Supplementary material for: Mutational profile of primary clear cell renal cell carcinoma predicts recurrence and potential candidacy for adjuvant immune checkpoint inhibition
Source: F1000Res. 2024 Jun 14;12:918. Originally published 2023 Aug 1. [Version 2] doi: 10.12688/f1000research.136087.2 (PMC11200057; doi:10.12688/f1000research.136087.2)
Supplement: Peer review history

# Reviewer report: Reviewer response for version 2

**DOI:** 10.5256/f1000research.163600.r291419

**Published:** 2024-06-25

**Authors:** Konstantinos Kamposioras

**Affiliations:** 1 Department of Medical Oncology, The Christie NHS Foundation Trust, Manchester, UK

The authors have dealt with the points raised in an appropriate manner.

Is the work clearly and accurately presented and does it cite the current literature?

Yes

If applicable, is the statistical analysis and its interpretation appropriate?

Yes

Are all the source data underlying the results available to ensure full reproducibility?

Yes

Is the study design appropriate and is the work technically sound?

Yes

Are the conclusions drawn adequately supported by the results?

Yes

Are sufficient details of methods and analysis provided to allow replication by others?

Yes

Reviewer Expertise:

Medical Oncology, Clinical biomarkers

I confirm that I have read this submission and believe that I have an appropriate level of expertise to confirm that it is of an acceptable scientific standard.

# Reviewer report: Reviewer response for version 1

**DOI:** 10.5256/f1000research.149223.r228073

**Published:** 2024-03-09

**Authors:** Athanasia Pavlopoulou

**Affiliations:** 1 Dokuz Eylül University, Izmir, İzmir, Turkey

The authors conducted a study of the molecular profile of RCC primary tumors in a small cohort of patients and examined whether particular gene mutations were associated with clinicopathological characteristics and outcomes of patients during a short follow up period. The following points need to be clarified:

 - how do the specific genes examined in this study relate to RCC prognosis in the literature?

 - the lack of transcriptional or epigenetic analyses should be discussed as a limitation of the study.

Is the work clearly and accurately presented and does it cite the current literature?

Yes

If applicable, is the statistical analysis and its interpretation appropriate?

Yes

Are all the source data underlying the results available to ensure full reproducibility?

Yes

Is the study design appropriate and is the work technically sound?

Yes

Are the conclusions drawn adequately supported by the results?

Yes

Are sufficient details of methods and analysis provided to allow replication by others?

Yes

Reviewer Expertise:

Molecular Biology; Computational Biology

I confirm that I have read this submission and believe that I have an appropriate level of expertise to confirm that it is of an acceptable scientific standard.

# Response

**Published:** 2024-03-10

**Authors:** Panagiotis J. Vlachostergios

**Affiliations:** Hematology & Medical Oncology, Cornell University, New York, New York, USA

**Reviewer 2**

 The authors conducted a study of the molecular profile of RCC primary tumors in a small cohort of patients and examined whether particular gene mutations were associated with clinicopathological characteristics and outcomes of patients during a short follow up period. The following points need to be clarified:

 1. How do the specific genes examined in this study relate to RCC prognosis in the literature?

Response: We appreciate the Reviewer’s comment. We have indicated in the Discussion section that: ”Inactivating mutations or/and low expression of these genes in primary renal tumors have been associated with poor outcomes. ^14 – 20^”

 2. The lack of transcriptional or epigenetic analyses should be discussed as a limitation of the study.

Response: We appreciate the Reviewer’s comment. We have now discussed the lack of transcriptional or/and epigenetic analyses as a limitation of our study, in the Discussion section.

 All edits in the revised manuscript are highlighted with ”Track changes” tool of MS Word.

# Reviewer report: Reviewer response for version 1

**DOI:** 10.5256/f1000research.149223.r192983

**Published:** 2023-08-22

**Authors:** Konstantinos Kamposioras

**Affiliations:** 1 Department of Medical Oncology, The Christie NHS Foundation Trust, Manchester, UK

I would like to congratulate the authors on their excellent work. However, there are a few points that need further explanation.

 The authors evaluated a number of genes in relation to clinical outcomes in a small number of cases.

 I would like to see the authors explain the biological rationale behind the two different molecular signatures and how this might relate to immunotherapy response, as suggested in the Discussion section. 

 I would also like to know how this study compares with previously published reports and if there is any consistency in the literature with regard to their findings.

 How do the authors see these findings changing or guiding clinical practice?

Is the work clearly and accurately presented and does it cite the current literature?

Yes

If applicable, is the statistical analysis and its interpretation appropriate?

Yes

Are all the source data underlying the results available to ensure full reproducibility?

Yes

Is the study design appropriate and is the work technically sound?

Yes

Are the conclusions drawn adequately supported by the results?

Yes

Are sufficient details of methods and analysis provided to allow replication by others?

Yes

Reviewer Expertise:

Medical Oncology, Clinical biomarkers

I confirm that I have read this submission and believe that I have an appropriate level of expertise to confirm that it is of an acceptable scientific standard, however I have significant reservations, as outlined above.

# Response

**Published:** 2024-03-10

**Authors:** Panagiotis J. Vlachostergios

**Affiliations:** Hematology & Medical Oncology, Cornell University, New York, New York, USA

**Reviewer 1**

 I would like to congratulate the authors on their excellent work. However, there are a few points that need further explanation.

 The authors evaluated a number of genes in relation to clinical outcomes in a small number of cases.

 1. I would like to see the authors explain the biological rationale behind the two different molecular signatures and how this might relate to immunotherapy response, as suggested in the Discussion section. 

Response: We appreciate the Reviewer’s comment. We have added a detailed discussion on the biological rationale of our study and how the selected genes may relate to response to immune checkpoint inhibition, as follows:

 “Cancer is a disease caused by the accumulation of genomic alterations. ^11^ This is a multistep process during which certain somatic mutations confer a survival advantage by activating signaling pathways that are associated with several hallmarks of cancer, including proliferative signaling, evasion of growth suppression, resistance to cell death, replicative immortality, angiogenesis, invasion and metastasis, reprogramming of energy metabolism and evasion of immune recognition and destruction. ^12^ Since these biological principles apply to the majority of solid tumors, including RCCs, we hypothesized that the higher the number of pathogenic mutations within a primary renal tumor, the more aggressive clinical behavior could be observed.

 The tumor suppressor VHL is the most frequently mutated gene in ccRCC and is a major player in renal cell carcinogenesis. However, VHL mutations alone are insufficient to drive disease progression, and have no prognostic or predictive value. ^13,14^ Mounting evidence has revealed an emerging role of other genes, heavily involved in chromatin rearrangement and epigenetic DNA modifications, including PBRM1, SETD2, BAP1, and KDM5C in ccRCC progression. ^15 , 16^ Inactivating mutations or/and low expression of these genes in primary renal tumors have been associated with poor outcomes. ^17 – 23^

 Interestingly, many of these frequently non-VHL genes, including PBRM1, BAP1, SETD2, have been investigated in the metastatic setting as potential predictors of response or resistance to immune checkpoint inhibitors (ICIs). For example, mutated PBRM1 was included in a composite biomarker together with tumor infiltrating lymphocytes (TILs) and absence of necrosis predicting a favorable response to ICIs. ^24,25^ Likewise, SETD2 loss results in greater vulnerability to immune checkpoint blockade compared to SETD2-proficient tumors, in vitro and in vivo. ^26^ BAP1-mutated tumors, while they portend a worse prognosis, they are more likely to be PD-L1 positive and demonstrate a more inflamed immune microenvironment suggesting that immune-targeting approaches could benefit these patients. ^27 ^Another DNA damage response and repair gene, ATM, was the second most frequently altered gene in ICI-responders with advanced RCC. ^28^ Pancancer analyses of tumors from ICI-treated patients suggested that those with KDM5C alterations have a substantially higher tumor mutational burden (TMB) level and a significantly higher level of CD8+ T cell infiltration and T effector signature which were associated with prolonged OS compared to the wild-type group. ^29^ Expression of PTEN/PI3K/mTOR pathway genes was significantly associated with numerous immune cells and immune-evasive mediators such as CD274/PD-L1, TGFBR1, CSF1R and PDCD1 in patients with ccRCC, ^30 ^suggesting that alterations in this pathway could also play a role in shaping these patients’ outcomes after treatment with ICIs.

 Our ongoing prospective study examined the mutational profile of patients with ccRCC on primary tumors after nephrectomy followed by observation or adjuvant immunotherapy with pembrolizumab and assessed for associations of mutated genes with high-risk features and DFS. We hypothesized that primary RCC tumors either umutated or harboring VHL mutations might be associated with a more benign clinical course after nephrectomy compared to those with a burden of mutations in other, non-VHL genes.“

*References (new):*

*11. Meyerson M, Gabriel S, Getz G: Advances in understanding cancer genomes through second-generation sequencing. Nat Rev Genet. 2010;11:685-696. 10.1038/nrg2841*

*12. Hanahan D, Weinberg RA: Hallmarks of cancer: the next generation. Cell. 2011; 144:646-674. 10.1016/j.cell.2011.02.013.*

*14. Kim BJ, Kim JH, Kim HS, Zang DY: Prognostic and predictive value of VHL gene alteration in renal cell carcinoma: a meta-analysis and review. Oncotarget. 2017;8:13979-13985. 10.18632/oncotarget.14704. PMC5355155.*

*24. Deutsch JS, Lipson EJ, Danilova L, et al: Combinatorial biomarker for predicting outcomes to anti-PD-1 therapy in patients with metastatic clear cell renal cell carcinoma. Cell Rep Med. 2023; 4:100947. 10.1016/j.xcrm.2023.100947. PMC9975323.*

*25. Wang N, Qin Y, Du F, Wang X, Song C: Prevalence of SWI/SNF genomic alterations in cancer and association with the response to immune checkpoint inhibitors: A systematic review and meta-analysis. Gene. 2022; 834:146638. 10.1016/j.gene.2022.146638.*

*26. Liu XD, Zhang YT, McGrail DJ, et al: SETD2 Loss and ATR Inhibition Synergize to Promote cGAS Signaling and Immunotherapy Response in Renal Cell Carcinoma. Clin Cancer Res. 2023; 29:4002-4015. 10.1158/1078-0432.CCR-23-1003. PMC10592192.*

*27. Kapur P, Rajaram S, Brugarolas J. The expanding role of BAP1 in clear cell renal cell carcinoma. Hum Pathol. 2023; 133:22-31. 10.1016/j.humpath.2022.07.022. PMC9898467.*

*28. Ged Y, Chaim JL, DiNatale RG, et al: DNA damage repair pathway alterations in metastatic clear cell renal cell carcinoma and implications on systemic therapy. J Immunother Cancer. 2020;8:e000230. 10.1136/jitc-2019-000230. PMC7311069.*

*29. Chen XJ, Ren AQ, Zheng L, Zheng ED: Predictive Value of KDM5C Alterations for Immune Checkpoint Inhibitors Treatment Outcomes in Patients With Cancer. Front Immunol. 2021;12:664847. 10.3389/fimmu.2021.664847. PMC8089485.*

*30. Li N, Chen J, Liu Q, et al: Prognostic significance and tumor-immune infiltration of mTOR in clear cell renal cell carcinoma. PeerJ. 2021;9:e11901. 10.7717/peerj.11901. PMC8378334.*

 2. I would also like to know how this study compares with previously published reports and if there is any consistency in the literature with regard to their findings.

Response:  We have added relevant literature that is in concordance with our study findings, as follows:

 “Our findings are in line with a previous work with slightly different design, whereby primary RCC tumors were segregated into VHL+0, VHL+1, VHL+2, and VHL+≥3 mutations.33 In both the discovery and validation cohorts of the study, those patients with a VHL+0 tumor had longer 5-year DFS and were proposed as candidates for surveillance. Conversely, patients with VHL+2 and VHL+≥3 tumors experienced shorter DFS rates of less than 50% and were deemed candidates for adjuvant therapy. ^33^”

*Reference (new):*

*33. Vasudev NS, Scelo G, Glennon KI, et al: Application of Genomic Sequencing to Refine Patient Stratification for Adjuvant Therapy in Renal Cell Carcinoma. Clin Cancer Res. 2023;29:1220-1231. 10.1158/1078-0432.CCR-22-1936. PMC10068441.*

 3. How do the authors see these findings changing or guiding clinical practice?

Response: We have now added a paragraph discussing the potential implications of our study for changing or guiding clinical practice, as follows:

 ”Collectively, our study combined with emerging evidence on the genomic landscape of RCC might open new avenues for both prognostication and better selection of a subgroup of patients with RCC that could benefit from adjuvant anti-PD1 immunotherapy.”
